# Biallelic variants in *ARHGAP19* cause a progressive inherited motor-predominant neuropathy

**DOI:** 10.1172/JCI184474

**Published:** 2025-10-14

**Authors:** Natalia Dominik, Stephanie Efthymiou, Christopher J. Record, Xinyu Miao, Renee Q. Lin, Jevin M. Parmar, Annarita Scardamaglia, Reza Maroofian, Simon A. Lowe, Gabriel N. Aughey, Abigail D. Wilson, Riccardo Curro, Ricardo P. Schnekenberg, Shahryar Alavi, Leif Leclaire, Yi He, Kristina Zhelcheska, Yohanns Bellaïche, Isabelle Gaugué, Mariola Skorupinska, Liedewei Van de Vondel, Sahar I. Da’as, Valentina Turchetti, Serdal Güngör, Gavin V. Monahan, Ehsan Ghayoor Karimiani, Yalda Jamshidi, Phillipa J. Lamont, Camila Armirola-Ricaurte, Haluk Topaloglu, Albena Jordanova, Mashaya Zaman, Selina H. Banu, Wilson Marques, Pedro J. Tomaselli, Busra Aynekin, Ali Cansu, Huseyin Per, Ayten Güleç, Javeria Raza Alvi, Tipu Sultan, Arif Khan, Giovanni Zifarelli, Shahnaz Ibrahim, Grazia M. S. Mancini, M.M. Motazacker, Esther Brusse, Vincenzo Lupo, Teresa Sevilla, A. Nazli Başak, Seyma Tekgul, Robin J. Palvadeau, Jonathan Baets, Yesim Parman, Arman Çakar, Rita Horvath, Tobias B. Haack, Jan-Hendrik Stahl, Kathrin Grundmann-Hauser, Joohyun Park, Stephan Zuchner, Nigel G. Laing, Lindsay A. Wilson, Alexander M. Rossor, James Polke, Fernanda Barbosa Figueiredo, André Pessoa, Fernando Kok, Antônio Rodrigues Coimbra-Neto, Marcondes C. Franca, Gianina Ravenscroft, Sherifa A. Hamed, Wendy K. Chung, Alan M. Pittman, Daniel P. Osborn, Michael Hanna, Andrea Cortese, Mary M. Reilly, James E.C. Jepson, Nathalie Lamarche-Vane, Henry Houlden

**Affiliations:** 1 Department of Neuromuscular Disease, UCL Queen Square Institute of Neurology, London, United Kingdom.; 2 Department of Anatomy and Cell Biology, McGill University, Montréal, Quebec, Canada.; 3 Cancer Research Program, Research Institute of the McGill University Health Center, Montréal, Quebec, Canada.; 4 Harry Perkins Institute of Medical Research, Center for Medical Research, University of Western Australia, Perth, Western Australia, Australia.; 5 Department of Epilepsy, UCL Queen Square Institute of Neurology, London, United Kingdom.; 6 Department of Brain and Behavioral Sciences, University of Pavia, Pavia, Italy.; 7 Institut Curie, Université PSL, Sorbonne Université, CNRS UMR3215, INSERM U934, Genetics and Developmental Biology, 75005 Paris, France.; 8 Translational Neurosciences, Faculty of Medicine and Health Sciences, and; 9 Laboratory of Neuromuscular Pathology, Institute Born-Bunge, University of Antwerp, Antwerp, Belgium.; 10 Department of Human Genetics, Sidra Medicine, Doha, Qatar.; 11 College of Health and Life Sciences, Hamad Bin Khalifa University, Doha, Qatar.; 12 Inonu University, Faculty of Medicine, Turgut Ozal Research Center, Department of Pediatric Neurology, Malatya, Turkey.; 13 Department of Molecular & Biomedical Sciences, City St George’s University of London, United Kingdom.; 14 Royal Perth Hospital, Perth, Western Australia, Australia.; 15 Molecular Neurogenomics group, VIB Center for Molecular Neurology, VIB, Antwerp, Belgium.; 16 Molecular Neurogenomics group, Department of Biomedical Sciences, University of Antwerp, Antwerp, Belgium.; 17 Department of Pediatric Neurology, Hacettepe University, Ankara, Turkey.; 18 Department of Medical Chemistry and Biochemistry, Medical University-Sofia, Sofia, Bulgaria.; 19 Department of Pediatric Neurology, Dr. M.R. Khan Shishu (Children) Hospital and ICH, Mirpur, Dhaka, Bangladesh.; 20 Department of Neurosciences, School of Medicine of Ribeirão Preto, University of São Paulo, São Paulo, Brazil.; 21 Clinical Hospital of Ribeirão Preto, Department of Neurosciences and Behaviour Sciences, University of São Paulo, Ribeirão Preto, Brazil.; 22 Department of Molecular Biology and Genetics, Biruni University, Istanbul, Turkey.; 23 Department of Pediatric Neurology, Faculty of Medicine, Farabi Hospital, Karadeniz Technical University, Trabzon, Turkey.; 24 Department of Pediatric Neurology, Erciyes University, Kayseri, Turkey.; 25 Children’s Hospital & the Institute of Child Health, Lahore, Pakistan.; 26 Neuropedia Children’s Neuroscience Center, Dubai, United Arab Emirates.; 27 Fakeeh University Hospital, Dubai, United Arab Emirates.; 28 Kids Neuro Clinic, Dubai, United Arab Emirates.; 29 CENTOGENE GmbH, Rostock, Germany.; 30 Department of Pediatrics and Child Health, Aga Khan University, Karachi, Pakistan.; 31 Department of Neurology, Erasmus MC University Medical Center, Rotterdam, Netherlands.; 32 Laboratory of Genome Diagnostics, Department of Human Genetics, Amsterdam UMC, University of Amsterdam, Amsterdam, Netherlands.; 33 Rare Neurodegenerative Diseases Laboratory, Centro de Investigación Príncipe Felipe (CIPF), Valencia, Spain.; 34 Hospital Universitari i Politècnic La Fe & IIS La Fe, Neuromuscular Diseases Unit, Department of Neurology, Valencia, Spain.; 35 Universitat de València, Valencia, Spain.; 36 Centro de Investigación Biomédica en Red de Enfermedades Raras (CIBERER), Madrid, Spain.; 37 Suna and İnan Kıraç Foundation, Neurodegeneration Research Laboratory (NDAL), Research Center for Translational Medicine (KUTTAM), Koç University School of Medicine, Istanbul, Turkey.; 38 Neuromuscular Reference Center, Department of Neurology, Antwerp University Hospital, Antwerp, Belgium.; 39 Neurology Department, Istanbul Faculty of Medicine, Istanbul University, Istanbul, Turkey.; 40 Department of Clinical Neurosciences, School of Clinical Medicine, University of Cambridge, Cambridge Biomedical Campus, Cambridge, United Kingdom.; 41 Department of Clinical Neurosciences, John Van Geest Centre for Brain Repair, School of Clinical Medicine, University of Cambridge, Cambridge, United Kingdom.; 42 Center for Rare Disease,; 43 Institute of Medical Genetics and Applied Genomics,; 44 Department of Epileptology, Center of Neurology, and; 45 Hertie Institute for Clinical Brain Research, University of Tübingen, Tübingen, Germany.; 46 John P. Hussman Institute for Human Genomics and; 47 Dr. John T. Macdonald Foundation Department of Human Genetics, University of Miami, Miami, Florida, USA.; 48 Centre for Neuromuscular Diseases, Department of Neuromuscular Diseases, UCL Queen Square Institute of Neurology, London, United Kingdom.; 49 Neurogenetics Laboratory, The National Hospital for Neurology and Neurosurgery and the North Thames Genomics Laboratory Hub, London, United Kingdom.; 50 Mendelics Genomic Analysis, São Paulo, São Paulo, Brazil.; 51 Universidade Federal Do Ceara - UFC and Hospital Infantil Albert Sabin, Fortaleza, Brazil.; 52 Department of Neurology, School of Medical Sciences, University of Campinas (UNICAMP), Campinas, São Paulo, Brazil.; 53 Department of Neurology, School of Medicine, Centro Universitário Uninovafapi – UNINOVAFAPI, Teresina, Piauí, Brazil.; 54 Department of Neurology and Psychiatry, Faculty of Medicine, Assiut University, Assiut, Egypt.; 55 Department of Pediatrics, Boston Children’s Hospital, Harvard Medical School, Boston, Massachusetts, USA.

**Keywords:** Genetics, Neuroscience, Neuromuscular disease

## Abstract

Charcot-Marie-Tooth (CMT) disease is a clinically and genetically heterogeneous group of hereditary neuropathies. Despite progress in genetic sequencing, for around a quarter of patients the disease has lacked a genetic explanation. Here, we identified 16 recessive variants in the RhoGTPase activating protein 19 gene (*ARHGAP19*) causing motor-predominant neuropathy in 25 individuals from 20 unrelated families. The ARHGAP19 protein acts as a negative regulator of the RhoA GTPase. In vitro biochemical and cellular assays revealed that patient variants impair the GTPase-activating protein (GAP) activity of ARHGAP19 and reduce ARHGAP19 protein levels. Through the use of patient lines, in vitro GAP assays and in silico molecular modeling, we provided evidence that CMT-associated ARHGAP19 variants act through a loss-of-function (LOF) mechanism. LOF in ARHGAP19 orthologues in *Drosophila melanogaster* and *Danio rerio* induced motor defects in axonal and synaptic morphology. Similar cellular phenotypes were observed in ARHGAP19 patient-derived motoneurons. Transcriptomic studies further demonstrated that ARHGAP19 regulates cellular pathways associated with motor proteins and the cell cycle. Taken together, our findings establish *ARHGAP19* variants as a cause of inherited neuropathy acting through a LOF mechanism.

## Abstract 2

We provide clinical, genetic and functional evidence that implicate ARHGAP19 as a novel causative gene for inherited neuropathy.

## Introduction

Charcot-Marie-Tooth (CMT) disease, also called hereditary motor and sensory neuropathy (HMSN), is the most prevalent Mendelian inherited neuropathy, varying in prevalence across populations but estimated on average to occur at a frequency of approximately 1 in 2,500 individuals (1).

Patients with CMT can range from mildly affected to severely disabled, and the disease presents with progressive distal wasting, weakness, and sensory loss often accompanied by foot deformity. Often, foot abnormalities such as *pes cavus* or hammer toes may be associated with the disease. Symptoms of CMT overlap between neuropathies and subtypes of CMT. Despite many genes being associated with inherited CMT, there remains a large proportion of genetically unexplained cases. However, family history, nerve conduction studies, and thorough clinical evaluation can aid differential diagnosis (2).

The Rho family of small GTPases is composed of 20 members that include RHOA, RAC1, and CDC42. Rho GTPases act as molecular switches by cycling between an inactive guanine nucleotide diphosphate–bound (GDP-bound) state and an active, triphosphate-bound (GTP-bound) state. They are involved in signaling pathways that control actin cytoskeleton reorganization, cell adhesion, migration, and cell division (3). The activity of Rho GTPases is tightly regulated by 3 classes of proteins: (a) Guanine nucleotide exchange factors (GEFs), which facilitate the exchange from GDP to GTP; (b) GTPase-activating proteins (GAPs), which stimulate the intrinsic GTPase activity, resulting in hydrolysis of GTP and protein inactivation; and (c) Guanine nucleotide dissociation inhibitors (GDIs), which sequester Rho GTPases and maintain their inactivity in the cytoplasm (4, 5). In humans, over 66 RhoGAPs and 80 RhoGEFs that act on members of Rho family have been identified, with many implicated in neurological disease, including CMT (5, 6). For example, mutations in *PLEKHG5*, encoding a GEF that regulates autophagy of synaptic vesicles in axonal terminals, cause recessive intermediate CMT (7), while variants in *MYO9B*, encoding a RhoGAP, have recently been shown to cause CMT type 2 (CMT2) and optic atrophy (8). Whether CMT can be caused by mutations in other regulators of Rho GTPases, however, has remained an open question.

**Figure 1:**
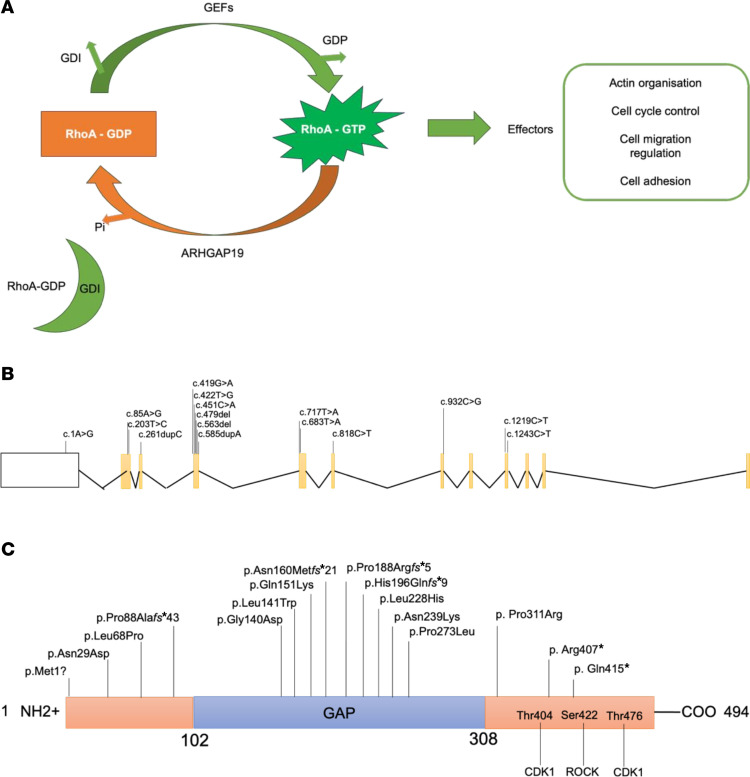
Schematic representation of ARHGAP19 and genetic findings. (**A**) Pathway showing GTPase-activating proteins (GAPs) such as ARHGAP19 responsible for promoting cycling of Rho GTPases between the active GTP-bound and the inactive GDP-bound conformations. GEF, Guanine nucleotide Exchange Factor; GDP, Guanine nucleotide diphosphate; GDI, Guanine Nucleotide Dissociation Inhibitor; Pi, dihydrogen phosphate. (**B** and **C**) Schematic diagrams of ARHGAP19 gene (**B**) and protein (**C**). Introns are not to scale. Exon numbers are according to the canonical transcript (NM_001136035). Amino acid changes are according to the reference sequence NP_001129507.

Here, we report the clinical phenotypes associated with biallelic, autosomal recessive variants in *ARHGAP19* in 25 individuals with CMT from 20 families. ARHGAP19 is a small RhoGAP protein, 494 amino acids (AAs) in length, which displays GAP activity towards RhoA but not Rac1 and Cdc42, thus acting as a negative regulator of the RhoA pathway (Figure 1A). ARHGAP19 has previously been shown to have an essential role in T-lymphocyte cytokinesis, and phosphorylation by kinases such as ROCK at Ser422, as well as CDK1, at Thr404 and Thr476 is essential for cell division (4, 9). Using patient cell lines, GAP assays, in silico molecular modeling, and *Drosophila melanogaster* and *Danio rerio* models, we show that CMT-associated ARHGAP19 variants cause progressive inherited motor-predominant neuropathy via LOF.

## Results

### Genetic findings.

**Figure 2:**
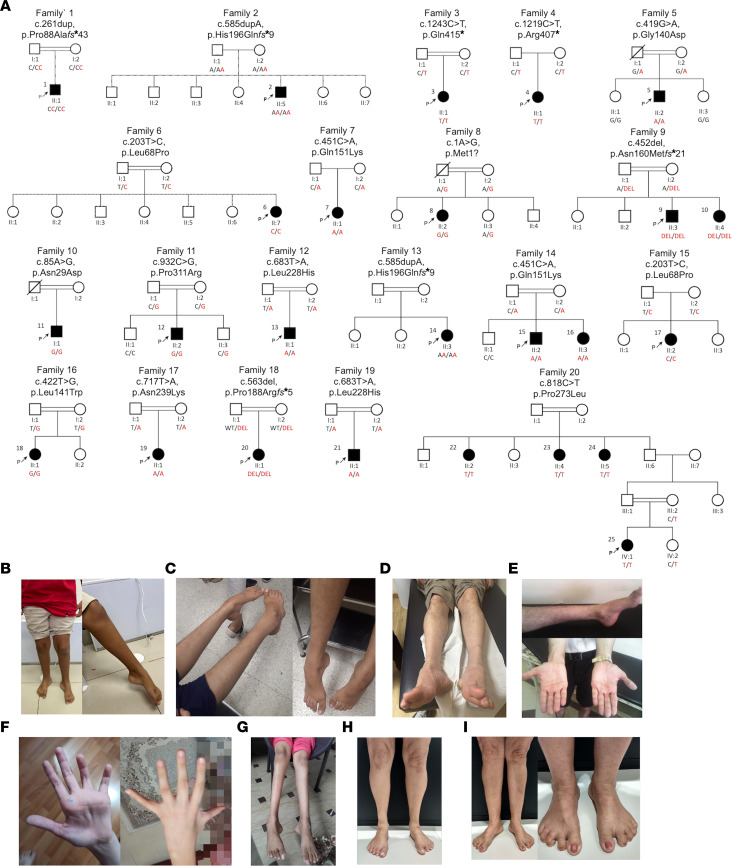
Genetic and clinical presentation of individuals harboring *ARHGAP19* variants. (**A**) Pedigrees of affected families showing segregation of the biallelic *ARHGAP19* variants identified. Clinical images presenting the spectrum of disease severity in (**B**) P1 (F1-II:1), (**C**) P3 (F3-II:1) (**D**) P10 (F9-II:4) (**E**) P2 (F2-II:5) (**F**) P17 (F15-II:3) (**G**) P20 (F18-II:1) (**H**) P22 (F21-II:2) and (**I**) P23 (F21-II:3), with predominant distal lower limb wasting, foot deformity, relatively mild thinning of intrinsic hand muscles and prominent sandal gap (except P17 (F15-II:3) who presented with acute left-hand weakness and still has upper limb–predominant disease).

We identified 20 unrelated families with 25 affected individuals harboring biallelic variants in *ARHGAP19* (Figure 1, B and C, and Figure 2A). All variant alleles identified in the families were either absent or observed only as heterozygous at extremely low frequencies in over 1.5 million alleles across multiple publicly available and private genetic variant databases (range 0–0.003) (Supplemental Table 2; supplemental material available online with this article; https://doi.org/10.1172/JCI184474DS1). Four variants (4/16, 25%) were observed in more than one family. Notably, p.His196Glnfs*9, p.Leu68Pro, p.Gln151Lys, and p.Leu228His variants were found in 2 independent Arab, 4 independent Turkish, and 2 independent Bangladeshi/Afghani families, respectively.

Haplotype plots of homozygous regions encompassing *ARHGAP19* variants were compared to assess whether the same causative variants were inherited from a common ancestor (Supplemental Figure 1). The chr10-97259559-A-T (p.Leu228His) and chr10:97263447-G-GT (p.His196Glnfs*9) variants are recurrent, since patients harboring the shared variants have different haplotypes. Patients F14-II:2 and F7-II:1 harbor the chr10-97263582-G-T variant (p.Gln151Lys) and share a large, 3.4 MB long, haplotype. MRCA analysis shows that this founder variant emerged about 26 generations ago (equivalent to 520 years). Variant chr10-97265979-A-G (p.Leu68Pro) is the other founder variant that we detected in patients F15-II:3 and F6-II:7 who harbor the same haplotype. The shared homozygous region is 3.0 MB, and they have inherited the variant from a common ancestor about 29 generations ago (equivalent to 580 years).

### Clinical features.

Tables 1 and 2 and Figure 2, B–I summarize the core clinical features of affected probands harboring *ARHGAP19* variants (Supplemental Table 1 for neurophysiology, Supplemental material for clinical vignettes). They originated from ancestral backgrounds including Pakistan, Turkey, Egypt, Syria, Bangladesh, Spain, Australia, Brazil, Iran, Dubai, and Afghanistan. 72% (18/25) were born from consanguineous parents. The mean (and median) age at symptom onset (AAO) was 9.9 (10.0) years, and at assessment was 22.8 (16.0) years. The presenting symptom was a motor deficit of the lower limbs in 91% (21/23), and 64% (14/22) had sensory involvement (symptoms or signs). As the disease progressed, symptoms typically remained either exclusively motor or motor predominant. Patients typically had a length-dependent pattern of lower motor neuron signs of areflexia and muscle atrophy, with foot drop. Lower limb–predominant disease (distal greater than proximal weakness, with normal upper limbs) was seen in 17% (4/23) and upper limb-predominant disease seen in 9% (2/23). Foot deformity was present in 80% (16/20), and high arches were frequent. However, the presence of brisk knee jerks and preserved lower limb reflexes each in 9% of the patient cohort (3/23), suggests some mild UMN involvement in these individuals. There were no consistent features outside of the peripheral nervous system.

A prominent feature of the phenotype is its significant asymmetry in terms of limb involvement, seen at onset or at assessment in 61% (14/23). Two cases also presented acutely with upper limb weakness on a background of mild or subclinical widespread neuropathy. Neurophysiology (Supplemental Table 1) was performed in 20 individuals. Detailed numerical study data were available in 16 of 20; 4 cases had a report only. All had a motor neuropathy, with variable sensory involvement. Evidence of motor conduction slowing was seen or described in 55% (11/20) and conduction block in 19% of the studies with numerical data (3/16). Combining clinical and neurophysiological data the following phenotypes were diagnosed: CMT-intermediate (CMTi) (2/25), CMT2 (7/25), hereditary motor neuropathy (HMN, 7/25), and CMT (indeterminate from neurophysiology, or no neurophysiology available, 9/25). The mean (median) ulnar motor velocity was 42.8 (45.0) m/s (range 25–58 m/s, *n* = 12).

It is difficult to comment on disease progression with only a single assessment available in many cases; however, in some individuals, from their clinical history, there is a more rapid progression than is typical for CMT. For example, 2 cases presented with acute upper limb weakness. The first (P13 (F12-II:1)), a male infant carrying the recurrent variant p.Leu228His presented with bilateral upper limb weakness over 15 days, with concurrent pneumonia. Spinal imaging was normal, but neurophysiology was never performed. Upper limb function improved over more than a year, but the patient sadly died at age 3 years. The second (P17 (F15-II:2)), a 13-year-old female carrying the recurrent p.Leu68Pro, presented with acute left-hand weakness, on a background of more widespread conduction slowing neuropathy. She was treated with intravenous immunoglobulin (Ig) for presumed chronic inflammatory demyelinating polyneuropathy (CIDP), with no clear response. Interestingly, P6 (F6-II:7), a female who carries the same homozygous variant, had walking difficulty from infancy but presented at age 12 years with a 4-month deterioration in left lower limb weakness. She had a conduction slowing neuropathy in the lower limbs. She is currently being treated with subcutaneous Ig for presumed CIDP.

### In silico modeling.

**Figure 3:**
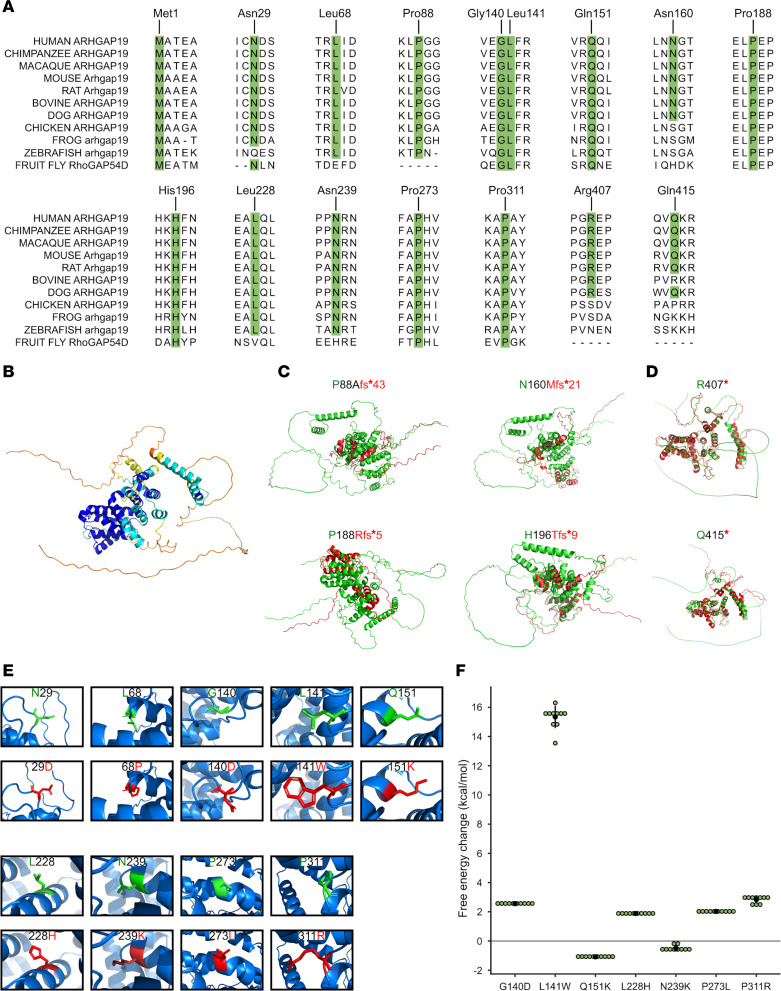
Investigating the effect of *ARHGAP19* variants in silico. (**A**) Multiple sequence alignment of ARHGAP19 orthologs shows species conservation of identified variants. (**B**) Predicted WT ARHGAP19 protein structure (UniProt: Q14CB8) using AlphaFold2, colored with the perresidue confidence score (pLDDT). Dark blue, very high confidence (pLDDT > 90); light blue, confident (90 > pLDDT > 70); yellow, low confidence (70 > pLDDT > 50); orange, very low confidence (pLDDT < 50). AlphaFold-generated mutant protein structures (red) derived from the identified frameshift (**C**) and nonsense (**D**) variants and aligned to the WT ARHGAP19 protein (green). (**E**) The effect of identified missense variants on 3D ARHGAP19 structure. WT residues are colored green and mutant residues are colored red. (**F**) Dot plots showing predicted free energy changes (ΔGMUT – ΔGWT) for missense variants on protein stability. Calculations were performed on variants within the very high confidence regions (pLDDT >90). Data are given as individual data points (green circle), as well as mean (black circle) ± SD (black line).

Multiple sequence alignments performed for ARHGAP19 orthologs across 11 animal species (Figure 3A), anchored to the human ARHGAP19 protein sequence, showed that most variants affect highly conserved amino acid residues. Of note, the variants affecting moderately conserved residues (Asn160, Arg407, and Gln415) are frameshift or nonsense. All variants segregated with disease within the families (Figure 3A). Computational variant prediction tools such as MutationTaster, SIFT, and PolyPhen, predict the functional impact of all but 2 variants as mainly damaging and deleterious. According to the American College of Medical Genetics and Genomics and the Association for Molecular Pathology (ACMG-AMP) system for variant classification, all the variants are classified as either likely pathogenic or pathogenic. The characteristics of all reported variants are summarized in Supplemental Table 2.

Three-dimensional visualizations of frameshift variants show substantial deviations from the WT protein (Figure 3, B and C), likely disrupting the RhoGAP domain structure. Nonsense substitutions display a similar structure to the WT protein, with greater variation only occurring in the final α-helix and the subsequent C-terminal end of the protein (Figure 3D). Missense substitutions showed little or no change. As such, the ‘mutagenesis’ function on PyMol was used to predict changes in protein structure and/or folding upon substituted amino acid residue incorporation into the sequence (Figure 3E). All substitutions showed changes in steric hindrance with nearby amino acid residues. The calculated free energy changes for p.Gly140Asp, p.Leu141Trp, p.Leu228His, p.Pro273Leu, and p.Pro311Arg substitutions show a decrease in free energy of greater than 1.6 kcal/mol, indicating a protein destabilizing effect (Figure 3F). Notably, 3 substitutions are predicted to result in protein instability with high confidence (p.Gly140Asp, p.Leu141Trp, and p.Pro311Arg). In addition, the p.Gln151Lys variant shows an increase in free energy, suggesting enhanced protein stability. Using further analysis with AlphaMissense, of the 9 missense variants identified, 7 (77.78%) are predicted to be likely pathogenic (Supplemental Table 4).

### Western blotting reveals significantly reduced ARHGAP19 in iPSC-derived motor neurons.

**Figure 4:**
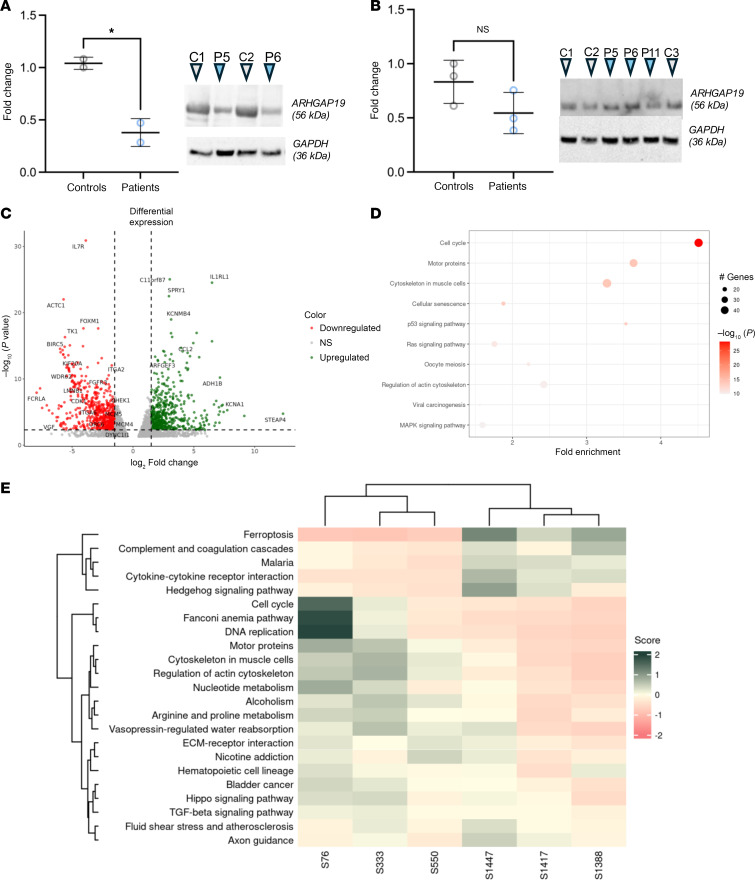
Western blot and RNA-seq analyses confirm downregulation of ARHGAP19 as well as cell cycle, motor, and muscular cytoskeleton pathways. (**A** and **B**) Protein expression levels of ARHGAP19 in iPSc-derived SMN lines (**A**) and patient-derived fibroblasts (**B**). Fibroblasts from P5 (F5-II:2), P6 (F6-II:7), and P11 (F10-II:1) harboring the c.419G>A (p.Gly140Asp), c.203T>C (p.Leu68Pro), and c.85A>G (p.Asn29Asp) variants, respectively, were analyzed by Western blot, as well as fibroblast-derived iPSc SMNs from P5 (F5-II:2) and P6 (F6-II:7) patients. (**C**) Volcano plot showing log_2_ of fold change in *ARHGAP19* mutants compared with controls and –log10 (adjusted *P*-value). (**D**) Pathway enrichment: differentially expressed genes with *P* value < 0.005 were selected for performing pathways enrichment analysis. Top 10 pathways with the highest enrichment scores were plotted, showing 3 cellular pathways highly enriched with dozens of affected genes and a very low *P*-value: cell cycle, motor proteins, and cytoskeleton in muscle cells. (**E**) Pathway scores per sample: enriched pathways were analyzed to compare their expression levels in patients and controls. Some pathways are overexpressed in patients while most of them are downregulated.

ARHGAP19 is expressed widely in human tissues, including the brain (Supplemental Figure 2) and shows a stable developmental expression in rat whole brain tissue and E18 dissociated cortical neurons (Supplemental Figure 3). Given the potential LOF in individuals harboring homozygous *ARHGAP19* variants, we investigated gene expression levels through qPCR and protein levels through Western blotting in fibroblasts harboring the c.85A>G (p.Asn29Asp) (P11), c.419G>A (p.Gly140Asp) (P5), c.203T>C (p.Leu68Pro) (P6) variants, and in fibroblast-derived iPSC motoneurons (MNs) of P5 (F5-II:2) and P6 (F6-II:7) (Figure 4, A and B; Supplemental Table 6). Whist *ARHGAP19* gene expression remained unchanged in both fibroblasts and motor neurons (data not shown), Western blotting assessment revealed significant reductions in ARHGAP19 protein levels in iPSC-derived motor neurons of patients compared with controls (Figure 4A).

### Transcriptional effects of ARHGAP19 variants.

To examine the effect of *ARHGAP19* variants on cellular pathways, we applied a transcriptomic approach on fibroblasts from patients who were ARHGAP19 deficient and people who were healthy controls. Differential expression analysis showed that there was no difference in ARHGAP19 mRNA levels between patient-derived fibroblasts and controls (log_2_ fold change = –0.29 and *P* value = 0.43). However, principal component analysis (PCA) showed a distinctive RNA expression pattern between patients and control tissue (Supplemental Figure 4). Differential expression and pathway enrichment analyses revealed that ARHGAP19 LOF alters the expression of genes linked to 3 cellular pathways: the cell cycle, motor proteins, and muscle cytoskeleton (Figure 4, C and D). Cellular pathways were further analyzed to infer their expression levels in patients compared with controls, which showed that the above-mentioned pathways are downregulated in patients with ARHGAP19 (Figure 4E).

### In vitro GAP assay shows GAP loss in ARHGAP19 variants located within the GAP domain.

**Figure 5:**
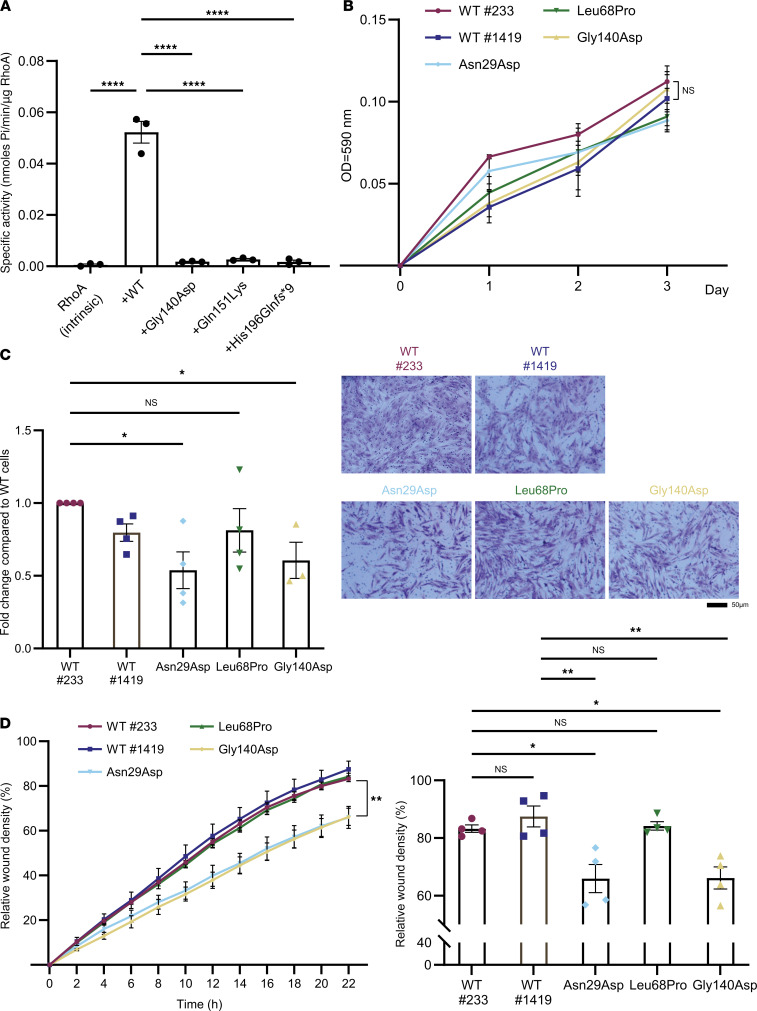
*ARHGAP19* variants have defective GAP activity and cell migration. (**A**) In vitro GAP activity assay measuring the GTPase rate of RhoA in the absence (intrinsic) or the presence of GAP domain from ARHGAP19 WT or mutants. Data are presented as means ± SEM from 3 independent experiments (*n* = 3; ****P < 0.0001; 1-way ANOVA) (**B**) MTT assays in fibroblasts from healthy controls (wt#223 and wt#1419) and ARHGAP19 mutants (*n* = 3). (**C** and **D**) Fibroblast cells were subjected to the Boyden chamber migration assay (**C**) or wound healing scratch assay as described in Methods (**D**) (*n* = 4). Representative images of migrated cells in the Boyden chamber assay are shown in **C**. Data are presented as mean ± SEM (*P < 0.05, **P < 0.01; 1-way ANOVA).

ARHGAP19 has been previously reported for its RhoGAP activity (4). Interestingly, several mutations in *ARHGAP19* cluster within the region encoding the GAP domain (Figure 1C). To investigate the GAP activity of these *ARHGAP19* variants, the GAP domain of ARHGAP19 or the mutated GAP proteins were expressed as GST fusion proteins in *E*. *coli* for in vitro GAP assays (Supplemental Figure 5). Specific GAP activity toward RHOA was measured as the rate of inorganic phosphate released by GTPase-mediated GTP hydrolysis. RHOA alone showed little intrinsic GTPase activity, while the addition of WT ARHGAP19 significantly accelerated the rate of RhoA-mediated GTP hydrolysis (Figure 5A). Two ARHGAP19 missense mutations, p.Gly140Asp, and p.Gln151Lys, abrogated the GAP activity of ARHGAP19, decreasing the GTPase hydrolysis rate to the basal level. Another ARHGAP19 mutation, p.His196Glnfs*9, which led to a truncated GAP domain of ARHGAP19, completely abolished the GAP activity, as evidenced by severely impaired phosphate release (Figure 5A). Altogether, these results demonstrate that these mutations in the GAP domain of ARHGAP19 are dominant LOF alleles, abolishing its GAP activity.

### Patient-derived fibroblast cells demonstrate impaired cell migration but not cell proliferation.

To further analyze the consequences of *ARHGAP19* variants on cellular morphology, we derived fibroblasts from the patients of family 5 (c.419G>A), family 6 (c.203T>C) and family 10 (c.85A>G), paired with age/gender matched healthy controls (WT).

Since the in vitro GAP assay indicated that *ARHGAP19* c.419G>A (p.Gly140Asp) shows defective GAP activity towards RhoA (Figure 5A), we hypothesized that variants in *ARHGAP19* may affect cell proliferation and migration. *ARHGAP19* variants and WT fibroblasts showed no significant differences in cell proliferation (Figure 5B). However, patient-derived fibroblasts carrying the p.Gly140Asp and p.Asn29Asp mutations had a significant reduction in cell migration compared with control cells using Boyden chamber migration and scratch assays (Figure 5, C and D). In contrast, patient-derived fibroblasts harboring the Leu68Pro showed no significant differences with control cells (Figure 5, C and D). Moreover, the cellular morphology of patient-derived fibroblasts carrying the Gly140Asp mutation was significantly more elongated compared with fibroblasts from healthy controls (WT #223 and WT #1419) (Supplemental Figure 6A), as quantified with the aspect ratio (length/width) (Supplemental Figure 6B). Fibroblasts from ARHGAP19 mutant proteins did not reveal increased stress fibers, but the elongated cell phenotype observed in fibroblasts from the ARHGAP19 Gly140Asp mutant protein suggest increased cell contractility and RhoA activity, similar to previous studies (4, 9). In addition, endogenous ARHGAP19 showed a strong nuclear localization in patient-derived fibroblasts (Supplemental Figure 6A), in agreement with previous studies showing nuclear localization of overexpressed ARHGAP19 (10, 11) and endogenous ARHGAP19 (4). Altogether, these results confirm the GAP-defective activity of the *ARHGAP19-* Gly140Asp mutant and suggest that the GAP function of the *ARHGAP19*-Asn29Asp mutant may also be altered.

### Patient fibroblast–derived iPSC MNs demonstrate increased branching but not length.

To further analyze the consequences of the ARHGAP19 variants, we interrogated the morphology of fibroblast-derived iPSC MNs carrying the Gly140Asp and the Leu68Pro mutations compared with those of age/gender matched healthy controls (WT) (Supplemental Figure 7, A and B). While we did not see any significant changes in neurite length (Supplemental Figure 7D), we observed increased neurite branching in the patient lines (Supplemental Figure 7C). Taken together with significant reduction of ARHGAP19 protein levels in iPSC-derived MNs (Figure 4A), these results may suggest that *ARHGAP19-* p.Gly140Asp and – p.Leu68Pro mutants may cause changes to the iPSC motor neuron morphology due to loss of function of ARHGAP19 protein.

### The ARHGAP19 ortholog RhoGAP54D regulates locomotion and motoneuron morphology in Drosophila.

To explore the consequences of ARHGAP19 LOF in vivo, we first utilized the fruit fly, *Drosophila melanogaster*. The *Drosophila* genome contains a single *ARHGAP19* ortholog termed *RhoGAP54D*, which exhibits 51% amino-acid similarity and 31% identity to the human ARHGAP19 protein, and is identified as the closest *Drosophila* orthologue of human *ARHGAP19* (and vice versa) by 12 different phylogeny-based bioinformatic programs (https://flybase.org/reports/FBgn0034249#orthologs).

**Figure 6:**
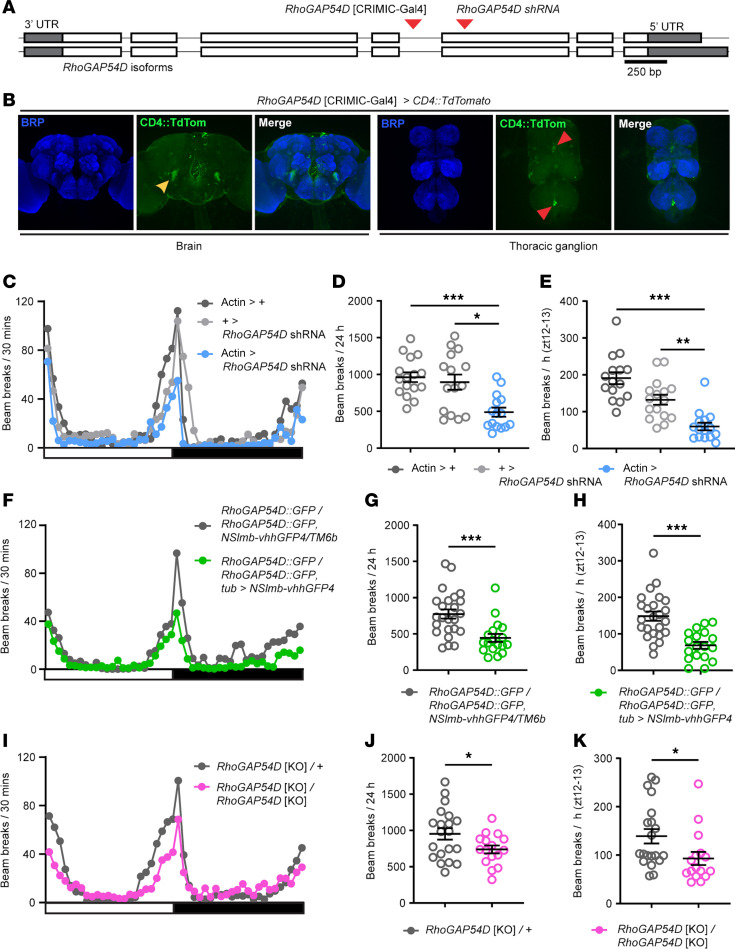
The *ARHGAP19* ortholog *RhoGAP54D* is important for *Drosophila melanogaster* locomotion. (**A**) Schematic of the *RhoGAP54D* locus. Exons, white blocks; untranslated regions (UTRs), grey blocks; intron, line. Insertion site of the *RhoGAP54D*[CRIMIC-Gal4] element and exonic region targeted by *RhoGAP54D* shRNA are shown (red arrows). (**B**) Confocal images illustrating *RhoGAP54D*-driven membrane-tagged CD4:TdTomato expression in the adult male *Drosophila* brain and thoracic ganglion. Brain, yellow arrow points to projections close to the antennal mechanosensory motor center. Thoracic ganglion, red arrows point to isolated cell bodies. Note the CD4:TdTomato signal surrounding Bruchpilot-labeled (BRP-labeled) neuropil domains. (**C**) Patterns of locomotor activity in flies globally expressing *RhoGAP54D* shRNA (*actin* > *RhoGAP54D* shRNA) and driver/shRNA alone controls. White bar, lights on; black bar, lights off. (**D** and **E**) Number of beam breaks across 24 hours (**D**) or during ZT12-13 (**E**), a period of peak activity. *n* = 15–16. (**F**–**H**) Patterns of locomotor activity (**F**), total (**G**) and peak (ZT12-13; (**H**) beam breaks in adult flies harboring a *RhoGAP54D* GFP fusion allele and expressing deGradFP components, enabling degradation of the RhoGAP54D:GFP fusion protein, and a *RhoGAP54D*:GFP homozygote control. *n* = 18 and 24 respectively. (**I**–**K**) Patterns of locomotor activity (**I**), total (**J**) and peak (ZT12-13; (**K**) beam breaks in adult flies heterozygous or homozygous for the *RhoGAP54D*[KO] null allele. *n* = 16 and 20 respectively. Central line in dot plots, mean. Error bars: SEM. *P < 0.05, **P < 0.005, ***P < 0.0005, 1-way ANOVA with Dunnett’s post hoc test (**D**), Kruskal-Wallis test with Dunn’s post hoc test (**E**), Mann-Whitney U-test (**G** and **K**), or *t* test with Welch’s correction (**H** and **J**).

To examine the expression of *RhoGAP54D* in the *Drosophila* nervous system, we utilized a CRIMIC T2A-GAL4 enhancer trap in intron 3 of the *RhoGAP54D* locus (12), which we term *RhoGAP54D*^CRIMIC-Gal4^ and that results in expression of Gal4 in the pattern of the endogenous gene (Figure 6A). Driving expression of a UAS-*CD4:TdTomato* reporter via *RhoGAP54D*^CRIMIC-Gal4^ allowed us to label *RhoGAP54D*-positive cells in the adult fly brain and thoracic ganglion (Figure 6B). In agreement with previously published single-cell RNA-seq data (13, 14) (Supplemental Figure 8A), this approach revealed sparse expression of *RhoGAP54D*, including projections to the antennal mechanosensory motor center (AMMC) and isolated cell bodies within the brain and thoracic ganglion (Figure 6B). In addition, we noted *RhoGAP54D*-driven TdTomato signal surrounding synaptic regions, suggesting that *RhoGAP54D* may be expressed in perineural and/or subperineural glia (SPG) that form the blood-brain barrier covering the central and thoracic neuropil domains (Figure 6B) (15).

Given that ARHGAP19 variants perturb movement in humans, we tested whether reducing RhoGAP54D expression disrupted locomotion in *Drosophila*. Since RhoGAP54D is likely expressed in a variety of cell-types, we used a global driver (*actin*-Gal4) in concert with an shRNA predicted to cleave *RhoGAP54D* mRNA in exon 3 of the primary transcript to induce ubiquitous RhoGAP54D knockdown (Figure 6A). Using quantitative PCR, we confirmed that expression of this shRNA caused an approximately 50% reduction in *RhoGAP54D* mRNA expression (Supplemental Figure 8B). We then used the *Drosophila* Activity Monitor (DAM) system (16) to test how reducing RhoGAP54D expression affected locomotor activity in adult flies (Supplemental Figure 8C). In 12-hour light: 12-hour dark conditions, WT *Drosophila* exhibit crepuscular peaks of activity centered around lights-on and lights-off, interspersed by periods of low activity (Figure 6C). Global RhoGAP54D knockdown reduced both total activity occurring over 24 hours and activity occurring in the hour following lights-off (zeitgeber time [ZT] 12–13), a measure of peak motor capacity (Figure 6, C–E). We extended this approach by inducing RhoGAP54D knockdown in all postmitotic neurons, motoneurons, muscle cells, glial cells, or neural stem cells (neuroblasts) (Supplemental Figure 9). No significant reductions in overall locomotor activity were detected following RhoGAP54D knockdown in these cell types, suggesting that RhoGAP54D is required in multiple cell types to influence locomotion in *Drosophila*.

To complement the above data, we utilized degradFP, a genetic system that promotes degradation of GFP-tagged fusion proteins via the ubiquitin pathway (17). Ubiquitous expression of degradGFP components in a background homozygote for a *RhoGAP54D:GFP* knock-in allele (18) similarly reduced overall and peak movement relative to *RhoGAP54D:GFP* homozygote controls (Figure 6, F–H). As a final confirmation, we generated a *RhoGAP54D* null allele (*RhoGAP54D*^KO^) through CRISPR-Cas9 gene editing (Figure 6, I–K). Comparison of *RhoGAP54D*^KO^ heterozygote and homozygote flies again revealed reduced overall and peak movement in *RhoGAP54D*^KO^ homozygotes relative to heterozygote controls (Figure 6, I–K).

Since ARHGAP19 LOF mutations cause motor neuropathy in patients, we asked whether *RhoGAP54D* knockout disrupts motoneuron function in *Drosophila*. We studied the larval neuromuscular junction (NMJ), an extensively studied model synapse (19). We observed reduced axonal length, reduced number of presynaptic boutons, and increased synaptic bouton area, in *RhoGAP54D*^KO^ homozygotes relative to heterozygote controls (Supplemental Figure 10, A–D). *RhoGAP54D*^KO^ homozygote larvae also showed clear locomotor deficits compared with heterozygote controls, consistent with impaired motoneuron function (Supplemental Figure 10, E and F). Thus, the ARHGAP19 orthologue RhoGAP54D promotes motoneuron development and function in *Drosophila*.

### Danio rerio arhgap19 is important for motor neuron function.

**Figure 7:**
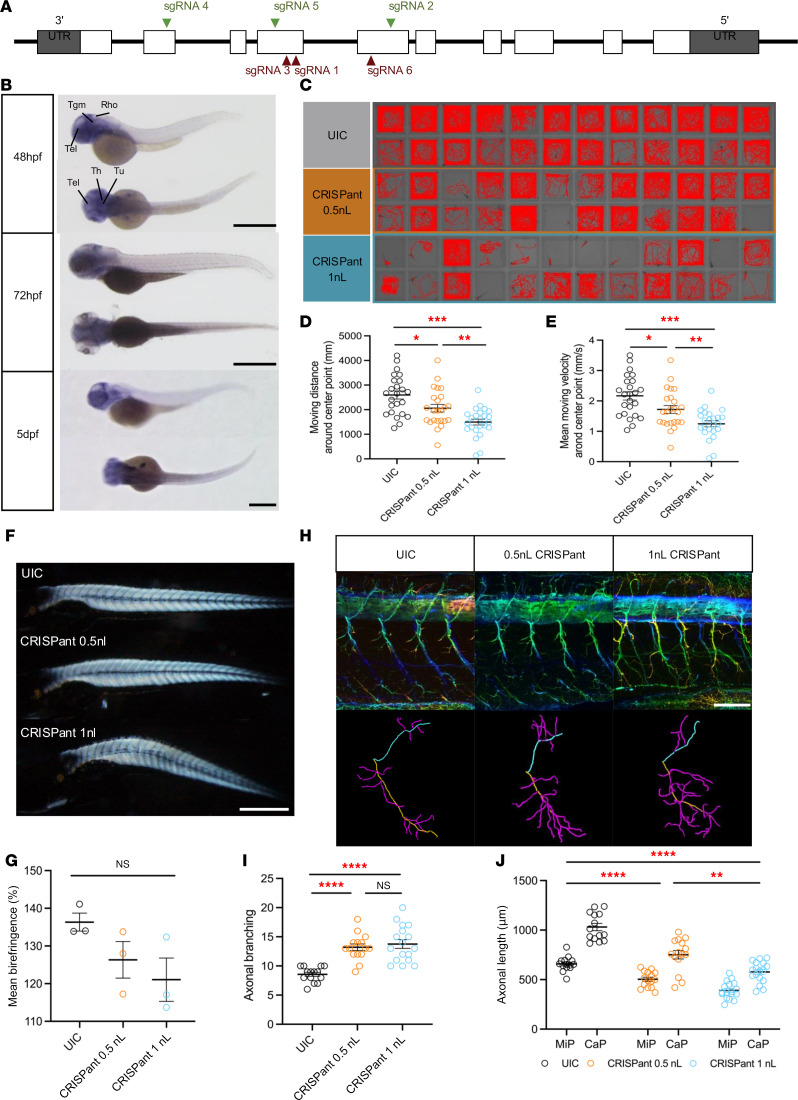
Zebrafish *Danio rerio* *arhgap19* is important for motor neuron function. (**A**) Schematic representation of 10 exons to cover the complete coding region. The position of the sgRNA targets are indicated (green illustrate the highest level of knockdown efficiency). (**B**) Zebrafish *arhgap19* expression at 3 different embryonic stages. At 48 hpf, WISH signal of *arhgap19* is localized in the forebrain and hindbrain regions; scale bar: 1,000 μm. Rho, Rhombencephalon (hindbrain); Tel, Telencephalon; Th, Thalamus; Tu, Tuberculum; Tgm, Tegmentum. (**C**–**E**) Behavior analysis of UIC and *arhgap19* mutant larvae at 5 dpf. **C** reveals the swimming trajectories of each larva. Quantification of total swimming distance (**D**) and swimming velocity (**E**) of UIC and *arhgap19* mutant zebrafish larvae for 30 minutes (UIC, CRISPant 0.5 nL, CRISPant 1 nL: *n* = 24). Each bar represents mean (± SEM). Asterisks above the bars indicate significant difference (*P ≤ 0.05, **P ≤ 0.01, ***P ≤ 0.001). (**F** and **G**) UIC and *arhgap19* mutant zebrafish larvae at 5 dpf were evaluated for muscle integrity using birefringence. (**F**) A representative image of one larva from each treatment group; scale bar: 1,000 μm. Each bar in plot **G** represents average birefringence (± SEM) for all zebrafish larvae (UIC, CRISPant 0.5 nL, CRISPant 1 nL: *n* = 3). (**H**–**J**) SMNs morphogenesis defects in *arhgap19* mutant zebrafish larvae. (**H**) Confocal imaging analysis and 3D reconstruction of SMNs in UIC and *arhgap19* mutant groups at 5 dpf; scale bar: 100 μm. (**I**) Axonal branching number of Cap axons in UIC and *arhgap19* mutant zebrafish larvae. (**J**) Average axonal length of Cap (yellow) and Mip (blue) axons in UIC and *arhgap19* mutant zebrafish larvae. Each bar represents mean (± SEM). Asterisks above the bars indicate significant difference (*P ≤ 0.05, **P ≤ 0.01, ***P ≤ 0.001, ****P ≤ 0.0001).

We next utilized zebrafish (*Danio rerio*) to examine the molecular, cellular, and developmental impact of ARHGAP19 LOF in a vertebrate system. To analyze the endogenous expression pattern and subcellular localization of zebrafish *arhgap19* during development, we conducted whole-mount in situ hybridization (WISH) assays at 3 different embryonic stages, utilizing digoxigenin-labeled antisense RNA probes specific for *arhgap19*. WISH analyses revealed a ubiquitous expression pattern of arhgap19 across multiple brain regions, notably in the forebrain and hindbrain compartments. This expression was predominantly enriched within neural tissues at 48 hours postfertilization (hpf). Specifically, heightened arhgap19 expression was observed in anatomically defined regions such as the cerebrum, thalamus, tuberculum, and tegmentum (Figure 7B). Intriguingly, a temporal downregulation of arhgap19 expression was evident as development progressed; by 5 days postfertilization (dpf), the expression levels had substantially diminished (Figure 7B).

To investigate the functional role of arhgap19 in neuronal and motor development, we generated an F0 biallelic knockout mutant model using CRISPR/Cas9 gene editing (Figure 7A). Off-target effects were evaluated by Sanger sequencing, which showed no sequence modifications among the top predicted off-target genes for each crRNA. Complementary RNA-seq analysis further supported these findings, as none of the candidate off-target genes exhibited significant changes in expression, providing additional confirmation that off-target effects were minimal (Supplemental Figure 11). To evaluate the changes in locomotor behavior in the arhgap19 mutant model, we collected 24 zebrafish larvae at 5 dpf and analyzed their motor swimming activity. Metrics such as the total duration of movement, aggregate distance traversed, and mean velocity were measured. Locomotor assays demonstrated that *arhgap19* knockout induced conspicuous motor deficits. Larvae in both mutant (genetically modified, referred to as CRISPants) groups exhibited decreased motor activity, alongside idiosyncratic and involuntary movements. In contrast, larvae from the Uninjected Control (UIC) group exhibited normal locomotor behavior, exploring the well’s periphery (Figure 7C).

Statistical analysis revealed significant discrepancy in motor parameters among the groups. Specifically, larvae from both CRISPants groups were significantly different in the total travel distance (Figure 7D; 1-way ANOVA, F(2, 69) = 13.954, and *P* < 0.0001, followed by Welch’s 2-tailed t test. CRISPant 0.5 nL, *t*(45.82) = –2.376 and *P* =.022; CRISPant 1 nL, *t*(41.939) = –5.418 and *P* < 0.0001). Likewise, the mean velocity also showed significant differences between mutant and control groups (Figure 7E; 1-way ANOVA, F(2, 69) = 13.954, and *P* < 0.0001, followed by Welch’s *t* test. CRISPant 0.5 nL, *t*(45.82) = –2.376 and *P* = 0.022; CRISPant 1 nL, *t*(41.939) = –5.418 and *P* < 0.0001). Larvae from the CRISPants 1nL group swam approximately twice as slow compared with those from the UIC group (Figure 7E). In addition, touch-evoked responses to physical stimulation were impaired in CRISPants of arhgap19 zebrafish (Supplemental Figure 12). Interestingly, *arhgap19* CRISPants injected with full dose showed a significantly shorter body length (Supplemental Figure 13A; 1-way ANOVA, F(2, 67) = 12.94 and *P* < 0.0001, followed by Welch’s 2-tailed *t* test with Bonferroni’s adjustment. CRISPant 1 nL, *t*(33.432) = –4.584 and ***P < 0.0001)

To investigate the impact of arhgap19 knockout on muscular architecture, we quantitatively assessed the birefringence intensity of zebrafish skeletal muscle at 5 dpf, employing a polarizing light stereomicroscope for imaging (Figure 7F). Statistical analysis of the birefringence levels revealed no significant difference between the control group and the 3 knockout groups (Figure 7G; 1-way ANOVA, F(2, 8) = 2.904, *P* = 0.131). These findings strongly suggest that skeletal muscle integrity remains largely intact in the absence of zebrafish arhgap19. Consequently, the motor deficits observed in the behavioral analyses are more likely attributed to impairments in motor neuron function rather than muscular deficiencies.

To investigate the effects of *arhgap19* knockout on primary motor neurons, we employed immunostaining techniques complemented by confocal microscopy for visualization. Notably, a more robust axonal bundle was observed in both CRISPant groups (Figure 7H). Quantitative assessments were carried out to analyze both axonal length and branching complexity. In *arhgap19* CRISPant larvae, the branching density of the Caudal Primary (CaP) Motoneurons was higher than that of the control group (Figure 7I; 1-way ANOVA, F(2, 43) = 21.341 and *P* < 0.0001, followed by Welch’s 2-tailed *t* test with Bonferroni’s adjustment. CRISPant 1 nL, *t*(22.271) = 6.33 and ***P < 0.001). Moreover, axonal length was markedly affected in *arhgap19* CRISPants. Statistical analysis revealed a notable reduction in the average length of CaP and Middle Primary (MiP) motoneurons in CRISPants, measuring 577.2 μm and 391.2 μm, respectively, in contrast with the control values of 1012.5 μm and 639.8 μm. (Figure 7J; 1-way ANOVA, F(2, 43) = 41.936 and *P* < 0.0001, followed by Welch’s 2-tailed *t* test with Bonferroni’s adjustment. CRISPant 0.5 nL, *t*(26.647) = –4.943 and ***P < 0.0001; CRISPant 1 nL, *t*(24.101) = –10.092 and ***P < 0.0001) Particularly, the axons of CaPs in *arhgap19* CRISPants exhibited diminished length, which failed to reach the ventral musculatures. Furthermore, *arhgap19* splice site and translation blocking morphants also exhibited branching abnormalities similar to those observed in CRISPants model (Supplemental Figure 13, D–F). Taken together, these results indicated that impaired motor neuron development was attributable to the loss of *arhgap19*.

To gain further insight into the role of *arhgap19* in motoneuron maturation, we carried out bulk RNA-seq on 48-hpf zebrafish larvae, comparing WT and *arhgap19* CRISPants larvae. Differential expression analysis confirmed a robust reduction of *arhgap19* transcript in the CRISPants larvae (log_2_FC = –1.99, adjusted *P* = 1.0 × 10^–11^). Overall, 38 genes met our significance threshold (FDR < 0.05), with 27 down regulated and 11 up regulated in the mutants. Gene-set enrichment showed that the up regulated gene cluster in proteasome function, cell-cycle control, and apoptotic pathways, whereas the down regulated genes are enriched for axonogenesis and neurotransmitter-receptor signaling. These findings suggest that loss of arhgap19 triggers stress- and proliferation-related programs while dampening axonal growth and synaptic-receptor pathways (Supplemental Figure 11).

## Discussion

Here, we show that biallelic *ARHGAP19* variants are a novel cause of inherited early-onset neuropathy. We identified 25 individuals harboring missense and nonsense biallelic variants both within and outside the functional GAP domain. The patients had a motor-predominant neuropathy with AAO almost exclusively in the first 2 decades of life. The clinical phenotype was generally length dependent, but there are some unusual features, including frequent conduction slowing ± conduction block, prominent asymmetry, subacute deterioration in some individuals, and upper limb onset. There was no observable genotype-phenotype correlation at the first examination; mean AAO of variants in GAP versus non-GAP domain 9.0 versus 11.6 years (*P* = 0.32, *n* = 15 and 8 respectively) and mean ulnar motor conduction velocity 41.7 versus 44.8 m/s (*P* = 0.65 *n* = 6 and 5). However, longitudinal studies would help better delineate the disease progression and any genotype-phenotype correlation.

Haplotype analysis using genetic data from our cohort as well as from control databases suggested that p.His196Glnfs*9 is unlikely to be from a recent common ancestor and possibly suggests 2 independent *ARHGAP19* mutational events within Arabian Middle Eastern populations. Moreover, p.Gln151Lys and p.Leu68Pro variants are founder effect variants, probably originating in Turkey. Variant p.Gln151Lys, which affects a highly conserved residue within the GAP domain, could have a significant structural or functional role. Additionally, this variant affects the same N-terminal catalytic stretch where the arginine motif at codon 143 is found and, therefore, might have a comparable mechanistic effect other *ARHGAP19* variants identified nearby, i.e. p.Gly140Asp and p.Leu141Trp. Together with p.Leu228His and p.Asn239Lys, they are predicted to disrupt the domain’s structure and subsequently its function as a GTPase-activating protein in a variety of cellular processes (4, 20). Similarly, previously reported variants in the Rho-GAP domain of Myo9b abrogate GAP activity (21). Interestingly, the corresponding *MYO9B* gene has recently been associated with a CMT2 subtype and isolated optic atrophy (8). Other variants, which increase free energy, such as p.Gln151Lys, may cause a deleterious effect to protein folding or stability through other mechanisms. We note that free energy changes were calculated using the AlphaFold2-derived ARHGAP19 protein. A recent study concluded that AlphaFold is not immediately applicable when calculating free energy changes for predicted protein structures (22). However, results were based on the first iteration of AlphaFold. Conversely, another study showed that free energy changes using AlphaFold2-predicted protein models consistently matched those of experimentally determined structures, particularly in high confidence regions (pLDDT > 90) (23). Thus, until an experimental ARHGAP19 protein structure is generated, it is important that in silico investigations of variant impact are complemented by corresponding functional studies.

The phenotype of conduction slowing in a motor-predominant neuropathy is unusual in CMT and limited to very few genes. Notably, recessive variants in the GEF gene *PLEKHG5* cause this phenotype and have been shown to cause loss of both large, myelinated fibers and thin myelination seen on nerve biopsy and motor axonal degeneration in a knockout mouse model (24, 25). Combined with the conduction slowing seen with biallelic variants in *ARHGEF10,* mechanistically, this would suggest that Rho GTPase activity, mediated by these GEF and GAP proteins, is implicated in a process that may involve both myelin and axonal pathologies.

Combined data from our in vitro and in vivo studies are consistent with this hypothesis (Figures 6 and 7, and Supplemental Table 9). Analyses of 3 global in vivo LOF *Drosophila* models and 3 CRISPR-Cas9 mediated knockout zebrafish models demonstrate a conserved role for ARGHAP19 orthologs in regulating locomotor activity and motoneuron morphology across metazoan species, supporting the above genotype-phenotype correlations in human patients harboring *ARGHAP19* mutations. In *arhgap19* mutant zebrafish, we observed more pronounced axonal bundles and a significantly increased number of axonal branches, suggesting that ARHGAP19 LOF may cause CMT, in part, by perturbing the cytoskeleton of motoneurons. Indeed, alterations in NMJ morphology of *RhoGAP54D* knockout *Drosophila* larvae are similar to those observed in mutants for presynaptic cytoskeletal proteins (26), and our transcriptomic analyses in patient-derived fibroblasts showed that pathways associated with motor proteins and the muscular cytoskeleton are downregulated by ARHGAP19 LOF, consistent with data showing that ARHGAP19 stimulates the intrinsically low GTPase activity of RhoA (27, 28), and that RhoA modulates cytoskeletal dynamics (27, 28) and axonal outgrowth and branching (29). Thus, one important avenue of future investigations will be to determine whether alterations in RhoA activity in motoneurons contributes to pathology in ARHGAP19-associated CMT.

However, we note that patient-derived MNs exhibit morphological changes only partially overlapped with phenotypes observed in zebrafish and *Drosophila* models (namely, an increase in axonal branching in patient-derived and zebrafish arhgap19 CRISPant MNs). Furthermore, the effects of cell-type–specific knockdown of the ARHGAP19 ortholog RhoGAP54D were inconsistent with a purely cell-autonomous requirement of RhoGAP54D in motoneurons for normal locomotion in *Drosophila*. Combined with the expression of ARHGAP19 and its orthologues in both neuronal and nonneuronal cell types (30–32), and, given that ARHGAP19 shows a stable developmental expression in rat whole brain from embryonic to postnatal stages, these data suggest that ARHGAP19 may be required from early on in multiple cell types, perhaps encompassing motoneurons and glial subtypes, to regulate motoneuron morphology and movement in vivo. Importantly, our zebrafish and *Drosophila* LOF alleles provide a platform for future studies to define the precise spatiotemporal requirements for ARHGAP19 in promoting motoneuron development and function across the lifespan.

How might *ARHGAP19* mutations perturb the cytoskeletal network in motor neurons? In this work, the pathogenicity of *ARHGAP19* variants p.Gly140Asp, p.Gln151Lys, and p.His196Glnfs*9 are supported by in vitro GAP activity assays, which show that variants within the GAP domain cause complete GAP loss. ARHGAP19 stimulates the intrinsic low GTPase activity of RhoA, thereby negatively regulating the RhoA/ROCK pathway. Our data showing that ARHGAP19 variants cause GAP loss suggest that these variants may affect the activity of RhoA, leading to ROCK activation, plausibly affecting downstream cellular pathways such as actin organization, cell migration, and axon outgrowth and guidance. Importantly, this is in line with in vitro data from patient-derived fibroblasts harboring p.Asn29Asp and p.Gly140Asp variants, which show significant decreases in cell motility and the increased neurite branching observed in iPSC MNs harboring p.Le0u68Pro and p.Gly140Asp variants.

However, further studies will be needed to directly assess the involvement of ROCK activation in ARHGAP19-associated CMT. Furthermore, we were only able to test a small subset of *ARHGAP19* variants where patient tissues were available, and we were not able to identify a clear correlation between the position of these variants and their effects on altered motility as a result of altered GAP activity, possibly due to alternative or compensatory mechanisms that may govern the relationship between GAP activity and cellular motility. Of note, variant p.Leu68Pro showed no change in the migration and wound healing assays, as opposed to WT controls. Interestingly, the 2 patients with this variant in our cohort were first suspected to have an autoimmune cause of neuropathy and were put on intravenous Ig for treatment of presumed CIDP with no improvement. These patients also have an upper limb involvement, and it is noteworthy that even if the variant lies outside the GAP domain, it is predicted to be pathogenic using various in silico methods.

Interestingly, protein expression analysis by Western blot revealed significant reductions in ARHGAP19 expression in iPSC motor neurons of patients compared with controls. It is noteworthy that decreased protein levels could contribute to functional deficits observed in the mutants with reduced GAP activity. While our current data do not allow us to definitively quantify the extent of this potential amplification, we acknowledge that a dual mechanism where lower overall protein abundance with decreased intrinsic GAP activity may better explain the observed cellular phenotypes. Taken together, our findings are consistent with the patients’ phenotype and functional assays, explaining that the muscular and motor defects observed in patients as well as animal models could be due to the dysregulation of the ARHGAP19-related signaling cascade. We provide evidence for a LOF mechanism as the pathomechanism of the disease and suggest that the LOF might have more severe consequences to motor neurons.

Our findings expand on previous work demonstrating ARHGAP19’s (33) involvement in Schwann cell development and myelination by identifying a new role in axonal processes. Yet, our current study does not fully elucidate the mechanism of axonal damage caused by ARHGAP19 deficiency and suggests noncell autonomous effect may be involved. Regarding the animal models created in this study, we were not able to investigate the expression of RhoGAP54D in the adult fly peripheral nervous system, and the zebrafish model showed no expression of the protein ortholog in its periphery. On the basis of frequent conduction slowing and the early onset phenotype observed, it will, therefore, be important to examine peripheral myelination in the human participants, as well as use better techniques for spatial localization and quantitative expression at different developmental stages in models of ARHGAP19 deficiency (34).

Despite the theoretically simple on/off switch model of Rho, the Rho GTPase signaling pathway has a more sophisticated picture. The high number of GAP and GEF proteins — 66 and 80 respectively, which outnumber Rho proteins — coupled with unclear specificity of these proteins, means that understating their signaling activities and roles in disease remains challenging. Nevertheless, overactive RhoA signaling in neurons, due to genetic variations or an imbalance between signaling molecules, has been reported in CMT disease (35). It is increasingly emerging that this is an important pathway not only in neuronal health but also in disease. Hence, modulation of this pathway may represent a potential strategy for future therapeutic treatments.

## Methods

### Sex as a biological variable.

Sex was not considered as a biological variable in this study.

### Study participants.

Individuals were recruited via an international collaborative network of research and diagnostic sequencing laboratories. Clinical data collection involved a detailed review of medical records, photographs, videos, and phone interviews, as well as a clinical reevaluation of nerve conduction studies by a neurologist. GeneMatcher and RD-Connect (GPAP) platform facilitated the identification of additional patients. Tables 1 and 2 and Supplemental Table 1 summarize the clinical details of the included cases.

### Next-generation sequencing.

Genomic DNA was extracted from peripheral blood of participants and parents according to standard procedures. Exome sequencing in subjects P2, P3, P12, P15, P18 was performed as described elsewhere (36) in Macrogen, Korea. Next-generation sequencing for other samples was carried out by referring centers (Supplemental Table 2). cDNA and protein sequence variants were described according to HGVS, using NM_032900.6 and NP_116289.4 as reference. Family segregation studies were performed via Sanger sequencing. Homozygosity mapping was performed on Automap (37) and haplotype analysis as previously described (38).

### Multiple sequence alignment.

To examine the conservation of substituted amino acid positions, we performed multiple sequence alignments of ARHGAP19 across multiple different species. ARHGAP19 protein sequences for each species were retrieved from UniProt using their respective accession codes (Supplemental Table 3). Alignments were performed using the MAFFT algorithm in Jalview (v2.11.2).

### Protein structure modelling and in silico mutagenesis.

The predicted WT ARHGAP19 protein structure (UniProt Q14CB8) was retrieved from the AlphaFold Protein Structure Database (39). 3D protein structures were visualized using PyMol (v.2.5.2). To examine variant effects, in silico mutagenesis for identified missense substitutions was performed through the PyMol ‘mutagenesis’ function. For nonsense and frameshift variants, mutant protein structures were generated using the open-source AlphaFold v2.0 (AlphaFold2) pipeline (39) with the input being FASTA files of the mutant amino acid sequences. The resultant protein structures were then aligned to the WT ARHGAP19 protein structure within PyMol. AlphaMissense substitution scores for identified *ARHGAP19* missense variants were obtained following Cheng et al. (40). To investigate the impact of *ARHGAP19* variants on protein stability, free energy changes between the WT and mutant proteins were calculated using FoldX v5.0. (41) Free energy change predictions were only performed on missense substitutions within high confidence regions (pLDDT > 90) of the AlphaFold2-predicted protein structure (23, 41–43).

### Generation of iPSCs and differentiation to SMNs.

Healthy control iPSCs were obtained through the *StemBANCC Consortium*. Patient-derived fibroblasts (P5 (F5-II:2) and P6 (F6-II:7)) were reprogrammed by nonintegrating Sendai viral vectors at Oxford StemTech. All iPSC lines were subject to quality control checks, including flow cytometry for pluripotency markers, global screening array karyotyping, and mycoplasma test. iPSCs were plated in Matrigel-coated 6-well plates and maintained in mTesR1 (StemCell Technologies). iPSCs were differentiated into Spinal Motor Neurons using a protocol previously published (44). Eleven days after seeding, cells were fixed for immunocytochemistry and harvested for RNA and protein extraction.

### Migration assay.

Migration assays were performed as previously described (45). Ten images were taken for each transwell insert using a Nikon inverted microscope (10 × objective lens, Nikon Eclipse TE300 Inverted microscope). Quantitative analysis was assessed using ImageJ. Data represent the fold change relative to WT cells obtained from at least 3 independent experiments.

### Scratch assay.

15,000 cells were seeded in 96-well plates and incubated for 24 hours. A scratch wound was made in the confluent cell monolayer of each well using the IncuCyte 96-well WoundMaker (Essen Bioscience) as described in manufacturer’s manual. After carefully removing the cellular debris, 100 mL of culture medium was added to each well. Cell images were captured every 2 hours using IncuCyte Live-Cell Imaging Systems (Essen BioScience, USA). Images were analyzed using the IncuCyte S3 software (2019A) to calculate cell confluency over time.

### RNA-seq from fibroblasts.

RNA-seq was performed on RNA from fibroblasts from participants P5 (F5-II:2), P6 (F6-II:7), and P11 (F10-II:1) as well as 3 controls using the Illumina TruSeq Stranded mRNA Library Prep and sequenced on the Illumina NovaSeq 6000. Analysis was performed as previously described (46–48).

### Protein expression and purification.

Recombinant WT and mutant GST-tagged ARHGAP19 GAP domain proteins were expressed in BL21 (DE3) bacteria and purified using glutathione-sepharose beads, as previously described (49). Eluted proteins were concentrated using Amicon Ultra-4 centrifugal filters (MilliporeSigma), resolved by SDSPAGE/Coomassie blue staining and quantified using bovine serum albumin (BSA) as standard.

### In vitro GAP assay.

The GAP activity of ARHGAP19 WT and protein mutants was assessed using the RhoGAP assay biochem kit (BK105, Cytoskeleton) according to manufacturer’s instructions. 1.5 μg of purified ARHGAP19 protein was mixed with His-RhoA protein and GTP for 20 minutes at 37°C. CytoPhos reagent was added for 10 minutes at room temperature before measuring the absorbance at 650 nm (Infinite M200 Pro Microplate reader, TECAN).

### Drosophila stocks and husbandry.

Flies were raised on standard fly food at 25°C in 12-hour light: 12-hour dark cycles. All experiments and crosses were conducted in these conditions unless otherwise specified. The following *Drosophila* stocks were obtained from the Bloomington Drosophila stock center: y[1] w[*]; P{Act5C-GAL4-w}E1/CyO (actin-Gal4; BDSC #25374), [1] *v[1]; P{y[+t7.7] v[+t1.8]=TRiP.HMS03522}attP40* (UAS-RhoGAP54D shRNA; BDSC #54051), y[1] w[*]; TI{GFP[3xP3.cLa]=CRIMIC.TG4.1}RhoGAP54D[CR02433-TG4.1]/SM6a (RhoGAP54D-Gal4; BDSC #92267), y[1] w[*]; P{w[+mC]=tubP-GAL4}LL7/TM3, Sb[1] Ser[1] (tubulin-Gal4; BDSC #5138), and w[*]; P{w[+mC]=UAS-Nslmb-vhhGFP4}3 (BDSC #38421). The RhoGAP54D:GFP fusion allele (w[*]; *RhoGAP54D:GFP:2Ma*; MKRS/TM6B) was generated via CRISPR-Cas9 gene editing, as described previously (18). The w[*]; P{Act5C-GAL4-w}E1/CyO insertion was outcrossed into an isogenized background (iso31) for 5 generations, with the X-linked *y*[1] marker removed in the process (Supplemental Table 7). Tissue-specific drivers were used to induce RhoGAP54D knockdown in all postmitotic neurons, motoneurons, muscle cells, glial cells, or neural stem cells (neuroblasts) (Supplemental Table 8).

### Generation of a Drosophila RhoGAP54D null allele.

The *RhoGAP54D*^KO^ LOF allele was generated using CRISPR/Cas9-mediated homologous recombination donor and guide plasmids to replace the *RhoGAP54D* reading frame from amino-acids (AA) T4 to L1004 (numbered according to the isoform RA, 1004 AA in length) by a *mw* gene flanked by 2 attP Phi31 recombination sites using the pCRISPR-del (50) and the pCFD5 (51) plasmids. pCFD5 was a gift from Simon Bullock, Medical Research Council Laboratory of Molecular Biology, Cambridge, United Kingdom (Addgene # 73914; RRID:Addgene_73914). To generate the pCRISPR-del-RhoGAP54D targeting the *RhoGAP54D* locus, the genomic 5′ HR1 (~1 kb) and 3′ HR2 (~1 kb) regions were PCR amplified using the following primers: HR1 5′- CCGGGCTAATTATGGGGTGTCGCCCTTCGCGGATCTCCGTAGACGCCGTTC and 5′- ACTCAAAGGTTACCCCAGTTGGGGCACTACTGCTTCCATGCAATCTGTGTGGTTTATCC, HR2 5′- ACTCAAAGGTTACCCCAGTTGGGGCACTACGACACGTTTAGCTTGGCCGCG and 5′-GCCCTTGAACTCGATTGACGCTCTTCTGTACAAGCCACCCCACACACTGAG to clone them in pCRISPR-del (underlined nucleotides matching the plasmid sequence used for cloning). We used 4 distinct gRNA, 2 in Nter and 2 in Cter to the *RhoGAP54D* locus, cloned in tandem in gRNA pCFD5 (Addgene #73914) plasmid to generate 2 pCFD5-RhoGAP54D guide plasmids using the following primer: 5- GCGGCCCGGGTTCGATTCCCGGCCGATGCAAGATTGCATGGAAGCAACGAGTTTTAGAGCTAGAAATAGCAAG and 5′-ATTTTAACTTGCTATTTCTAGCTCTAAAACTTTCGCCCGGATACTTCTCGTGCACCAGCCGGGAATCGAAC as well as 5′-GCGGCCCGGGTTCGATTCCCGGCCGATGCAAGATTGCATGGAAGCAACGAGTTTTAGAGCTAGAAATAGCAAG and 5′-ATTTTAACTTGCTATTTCTAGCTCTAAAACTATCCATCGTTGCTTCCATGTGCACCAGCCGGGAATCGAAC-. Cloning was performed by primer annealing and ligation, or by SLIC. All regions amplified by PCR as well as junctions between tag and genomic sequences were checked by sequencing. *RhoGAP54D*^KO^ allele was then generated by coinjection of the pCRISPR-del- RhoGAP54D (300 ng/ml) and each of the guide plasmid (25 ng/ml) in embryos of the PBac{y+-attP-9A}VK00027 (BDSC #51324) line. Embryo injection was performed by BestGene transgenesis services. The RhoGAP54D^KO^ genomic deletion and locus organization were confirmed by PCR and sequencing.

### Drosophila activity assays.

To quantify locomotion in adult male flies, the *Drosophila* Activity Monitor (DAM; Trikinetics inc.) was used as described previously (16).

### Drosophila IHC.

Adult brains were dissected and immuno-stained as described previously (52). Primary antibodies were as follows: mouse anti-Bruchpilot (BRP) (nc82, Developmental Studies Hybridoma Bank), 1:50; rabbit anti-dsRed (Clontech, #632496), 1:1000. Secondary antibodies were: goat anti-rabbit AlexaFluor-555 (ThermoFisher, #A32732), 1:1000; and goat anti-mouse AlexaFluor-647 (ThermoFisher #A21236), 1:500. Brains were incubated in primary and secondary antibodies overnight at 4°C. Brains were washed then mounted and imaged in SlowFade Gold anti-fade mounting solution (ThermoFisher Scientific). Images were taken using a Zeiss LSM 710 confocal microscope with an EC ‘Plan-Neoflar’ 20 × air objective. Images were analyzed using ImageJ.

### qPCR using Drosophila tissue.

Total RNA was extracted from dissected brain tissue of *actin* > UAS-*RhoGAP54D* shRNA and *actin* > UAS-*mCherry* shRNA (control) adult male flies using standard phenol chloroform extraction. 0.5 μg of total RNA was reverse transcribed to generate cDNA (Superscript III, Thermofisher). Technical duplicates of the qPCR samples were prepared with Fast SYBR Green Master Mix (Thermofisher), using 500 nM of each primer (forward: ATGGAAGCAACGATGGATACG; reverse: CTCGTGACAGGGGAGATCGAA), and 1 μl of the reverse transcription reaction. qPCR was performed using the QuantStudio 3 Real-Time PCR System (Thermo Fisher Scientific). The PCR conditions included a prerun at 95°C for 5 minutes, followed by 40 cycles of 30 seconds at 95°C, 30 seconds at 58°C, and 45 seconds at 72°C. PCR amplification specificity was determined by melting curve analysis with a range from 60°C to 95°C. The values of the cycle threshold (ΔCT) of the target mRNAs were normalized to the mRNA of RpL4 (forward primer: TCCACCTTGAAGAAGGGCTA; reverse primer: TTGCGGATCTCCTCAGACTT) using QuantStudio Design&Analysis Software (Thermo Fisher Scientific) and expressed as a fold change relative to *actin* > UAS-*mCherry* shRNA controls.

### Danio rerio functional analyses.

WT *Danio rerio* were housed and bred within UCL and Sidra Medicine Fish Facilities at 28.5°C on a 14-hour day/10-hour dark cycle. For WISH, zebrafish embryos were fixed in 4% paraformaldehyde (PFA/PBS) overnight at 4°C, dechorionated, and dehydrated in methanol at –20°C. A T7 bacteriophage promoter containing amplicon of *arhgap19* was synthesized from cDNA libraries at 3 different developmental stages: 24 hpf, 48 hpf, and 5 dpf. A primer set was designed with the T7 bacteriophage promoter sequence incorporated at the 5′ end of the reverse primer for antisense probe production (Forward primer: 5′-GGCCGAATTCTCACAGCTAC-3′; Reverse primer: 5′-TAATACGACTCACTATAGGTCTTACACGCGCTGATGAAC-3′). A Digoxigenin-labeled antisense probe was synthesized using a DIG-RNA Labeling Kit (T7 polymerases, Roche). WISH was carried out as previously described (53) on 48 hpf, 72 hpf, and 5 dpf larvae, with NBT/BCIP Stock Solution (Roche) in the staining buffer. The stain was fixed in methanol and embryos were mounted in 3% methylcellulose for imaging.

The zebrafish arhgap19 gene (ENSDARG00000083189) is an ortholog of *Homo sapiens* ARHGAP19; there is 59.95% nucleotide similarity and 55 % amino acid similarity between the zebrafish and human loci. No zebrafish paralogs corresponding to ARHGAP19 exist. CRISPR/Cas9-mediated F0 biallelic knockout was performed as described previously (54) (Supplemental Table 5) (55). NGS sequencing showed that sgRNAs 2, 4, and 5 manifested high gene-editing efficiency, with respective average modification rates of 96.1%, 99.7%, and 94.6%. Conversely, sgRNA 3 exhibited suboptimal performance, achieving a comparatively low modification rate of merely 11%.

An antisense translational blocking Morpholino (MO, GeneTools.LLC) against the AUG-containing mRNA sequence (5′-GGCCATCTTTCATCTTCCGTTTGAA-3′) and a splice MO targeting the Exon1-Intron1 (E1I1) boundary (5′-ATAAATCTTCGTTACCTTCTGTCTC-3′) was designed to knockdown arhgap19. MOs were diluted to the desired working concentrations (3 ng, 4 ng, 6 ng, and 8 ng per embryo) before use. Microinjection was performed by injecting 0.5–1nL morpholino solution into 1 to 2-cell stage embryos in the yolk. Dose-dependent phenotyping was used to identify an appropriate concentration that balanced survival with specific phenotypic changes.

### Danio rerio behavioral assays.

Zebrafish larvae at 5 dpf were transferred into individual wells of a multiwell plate. Baseline locomotor activity was recorded for 30 minutes and analyzed using the DanioVision (Noldus, Netherlands) monitoring chamber, integrated with the EthoVision XT 14 video tracking software (Noldus, Netherlands). Plots were analyzed for distance traveled (in millimeters) and velocity. For assessment of muscle integrity, zebrafish larvae at 5 dpf were fixed and their skeletal muscle was analyzed for total birefringence using polarized light microscopy (Nikon SMZ100). Touch-evoked response assay is performed by stimulating swimming via the physical stimulation of larvae at 96 hpf, control group *n* = 20 and CRISPant group *n* = 40. Response is recorded using a high-speed camera as previously described (56).

### Danio rerio IHC.

Zebrafish larvae at 5 dpf were permeabilized with 10 μg/ml proteinase in PBS K in PBS Tween (0.2%, PBT), followed by 20 minutes fixation in 4% PFA. Larvae were blocked with goat serum (5% in PBT) and incubated with antitubulin mouse monoclonal antibody (1:500 dilution; T6793-2ML, Sigma) overnight at 4°C. After 4 PBT washes, larvae were incubated with Alexa Fluor anti-mouse 568 secondary antibody (1:1000 dilution; A-11004, ThermoFisher) in the dark at 4°C. For nucleus detection, larvae were incubated with DAPI (1:1000 dilution; D1306, ThermoFisher) under dark conditions at 4°C. Embryos were washed again in PBT (4 times) and imaged using a Nikon A1R confocal microscope to assess motor neuron morphology. Confocal fluorescent images were processed and code depth adjusted with FIJI/ImageJ while motor neuron quantification was conducted using the SNT plugin.

### Statistics.

Statistical evaluations were carried out using IBM SPSS Statistics. One-way ANOVA and Welch’s *t* test were used for statistical comparisons between groups with calculated SD/SEM. For *Drosophila* work, we ran nonparametric tests for normal distribution using Shapiro-Wilk test for normality. Normally distributed datasets were subject to tests as stated. Nonnormally distributed datasets were subject to Mann-Whitney U-test or Kruskal-Wallis test with Dunn’s post hoc test (single or multiple comparisons respectively). Significant differences were determined at a threshold of *P* < 0.05. The corresponding *P*-values are provided in the Figure legends.

### Study approval.

Samples and clinical information were obtained, with informed consent, using local institutional review board (IRB, Sponsor EDGE ID: 146653 IRAS: 310045) ethics for functional analysis of human DNA, fibroblasts and biomaterial. All experiments were conducted under licenses awarded by the UK Animal (Scientific Procedures) Act of 1986 implemented by the Home Office in England and Qatar Ministry of Public Health guidelines.

### Data availability.

The data that supports the findings of this study are available within the paper and in the supplemental material. Whole-exome sequencing data are not publicly available due to privacy or ethical restrictions. The identified *ARHGA19* variants were submitted to the LOVD database (https://databases.lovd.nl/shared/genes/ARHGAP19), with the LOVD variant IDs: #0000971425, #0000971426, #0000971427, #0000971440, #0000971442, #0000971444, #0000971446, #0000971450, #0000971466, #0000971468, #0000971470, #0000971471, #0000971473, #0000971474, #0000971475, #0000971478, #0000971479 and #0000971480.

## Author contributions

ND, SE, JECJ, DPO, and NLV conceived of and designed the study. ND, XM, RQL, RC, RPS, LL, YH, and SID conducted experiments. ND, RQL, XM, LL, YH, CJR, and SA analyzed data. YB and IG provided reagents. ND, SE, and RQL wrote the original draft of the manuscript. DPO, A Cortese, MMR, JECJ, NLV, and HH provided critical revisions to the manuscript. A Cansu, A Cakar, A Cortese, ADW, AG, AJ, AK, AMP, AMR, ANB, AP, ARCN, AS, BA, CAR, CJR, DPO, EB, EGK, FBF, FK, GMSM, GNA, GR, GVM, GZ, HH, HP, HT, IG, JB, JECJ, JHS, JMP, J Park, J Polke, JRA, KGH, KZ, LAW, LL, LW, MCF, MH, MMM, MMR, MS, MZ, ND, NGL, NLV, PJL, PJT, RC, RH, RJP, RM, RPS, RQL, SAH, SA, SAL, SE, SG, SHB, SID, SI, ST, SZ, TBH, T Sultan, T Sevilla, UP, VL, VT, WKC, WM, XM, YB, YH, and YJ acquired data and reviewed and approved the final version of the manuscript. The authorship order among the co–first authors was determined to reflect their substantial contribution to the study, while acknowledging varying degrees of involvement in the collaborative work.

## Funding support

This work is the result of NIH funding, in whole or in part, and is subject to the NIH Public Access Policy. Through acceptance of this federal funding, the NIH has been given a right to make the work publicly available in PubMed Central.

- The Wellcome Trust Strategic award (Synaptopathies) (WT093205 MA and WT104033AIA) to the SYNaPS Study Group collaboration.
- MRC strategic award to establish an International Center for Genomic Medicine in Neuromuscular Diseases (ICGNMD) MR/S005021/1 (ND, SE, CJR, PJT, MGH, MMR, and HH received direct support from this award).
- National Institute for Health Research University College London Hospitals Biomedical Research Center (NIHR UCL BCR).

## Supplementary Material

Supplemental data

Unedited blot and gel images

Supplemental table 8

Supplemental table 9

Supporting data values

## Figures and Tables

**Table 2:**
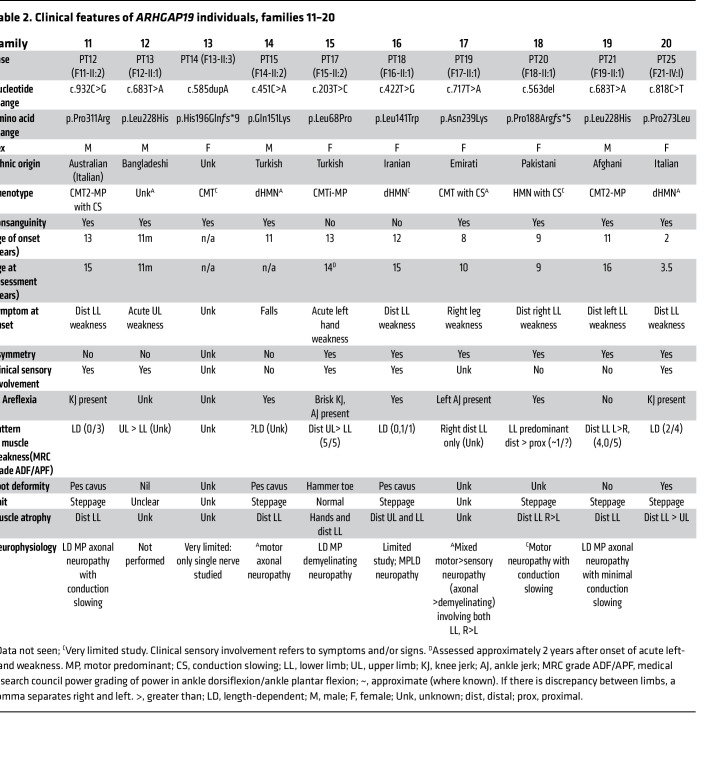
Clinical features of *ARHGAP19* individuals, families 11–20

**Table 1:**
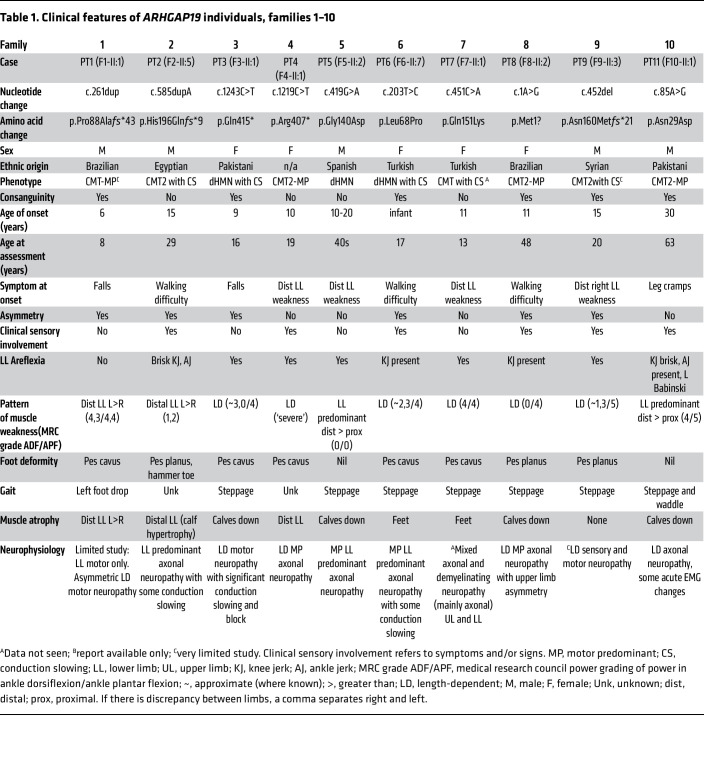
Clinical features of *ARHGAP19* individuals, families 1–10

## Version 1 10/14/2025

In-Press Preview

## Version 2 12/01/2025

Electronic publication
